# Latent Growth Curve Models for Biomarkers of the Stress Response

**DOI:** 10.3389/fnins.2017.00315

**Published:** 2017-06-06

**Authors:** John M. Felt, Sarah Depaoli, Jitske Tiemensma

**Affiliations:** Department of Psychological Sciences, University of California, MercedMerced, CA, United States

**Keywords:** latent growth curve model, stress response, cortisol, alpha-amylase, biomarkers

## Abstract

**Objective:** The stress response is a dynamic process that can be characterized by predictable biochemical and psychological changes. Biomarkers of the stress response are typically measured over time and require statistical methods that can model change over time. One flexible method of evaluating change over time is the latent growth curve model (LGCM). However, stress researchers seldom use the LGCM when studying biomarkers, despite their benefits. Stress researchers may be unaware of how these methods can be useful. Therefore, the purpose of this paper is to provide an overview of LGCMs in the context of stress research. We specifically highlight the unique benefits of using these approaches.

**Methods:** Hypothetical examples are used to describe four forms of the LGCM.

**Results:** The following four specifications of the LGCM are described: basic LGCM, latent growth mixture model, piecewise LGCM, and LGCM for two parallel processes. The specifications of the LGCM are discussed in the context of the Trier Social Stress Test. Beyond the discussion of the four models, we present issues of modeling nonlinear patterns of change, assessing model fit, and linking specific research questions regarding biomarker research using different statistical models.

**Conclusions:** The final sections of the paper discuss statistical software packages and more advanced modeling capabilities of LGCMs. The online Appendix contains example code with annotation from two statistical programs for the LCGM.

## Latent growth curve models for biomarkers of the stress response

The stress response is a complex, dynamic process. This process can be best characterized as a negative emotional experience accompanied by predictable biochemical, physiological, and behavioral changes that are relevant to adaptation (Lazarus and Folkman, 1984; Baum, 1990; Dougall and Baum, 2012). Many methods have been developed to evaluate the stress response using self-reported (i.e., subjective) measures and biomarkers (i.e., objective). Self-report measures and biomarkers are collected over time, and therefore require alternative statistical methods that can handle issues of repeated measurements. However, there are many methodologies that stress researchers can choose from, depending on the properties of the data and research questions.

The methodological approaches used within stress research have evolved over time; specifically, the approaches used to evaluate the change in biomarkers. The stress research literature, particularly relating to biomarkers, has commonly incorporated methods such as mean-difference-based approaches (see e.g., Brouwer and Hogervorst, 2014; Alsalman et al., 2016; Kempke et al., 2016; Gerber et al., 2017) and mixed effects models (see e.g., Saxbe et al., 2008; Van Lenten and Doane, 2016). However, the field has recently started to incorporate applications of latent variable modeling (LVM) to examine the stress response. This approach, although longstanding in other fields (e.g., Education or Economics), is relatively new to the stress response literature—with only some very recent applications (e.g., Hagger-Johnson et al., 2010; Thornton et al., 2010; Giesbrecht et al., 2015). The incorporation of more advanced statistical methods such as LVMs represents an evolution of methodological approaches that correspond to the development of more sophisticated research questions being asked within biomarker-related inquires.

## Purpose of manuscript

The purpose of this manuscript is to expand upon the most commonly used statistical methods in stress research with biomarkers. An alternative, powerful modeling framework (i.e., the latent growth modeling framework) will be discussed in the context of specific research questions generated from commonly used laboratory stressors. The paper includes a comparison of statistical software for estimating LVMs. Sample code for various statistical software programs will be provided in the online Appendix for each LVM discussed^1^.

## Traditional methods

Mean-difference-based approaches, such as analysis of variance (ANOVA) and multivariate ANOVA (MANOVA), are useful when researchers are interested in evaluating average change over time (Hedeker and Gibbons, 2006a,b). However, ANOVA and MANOVA approaches are limited in the types of questions that can be answered. Researchers interested in evaluating the individual differences observed in the stress response (e.g., Schlotz et al., 2011; Skoluda et al., 2015) should move to an individual-difference-based approach. This approach typically implements hierarchical linear models (also called multilevel models), which include mixed regression models (MRMs). MRMs provide insight into average change over time while modeling individual variation through the specification of random effects—or the estimation of an intercept and any number of slopes (Hedeker and Gibbons, 2006c). While MRMs also represent a flexible modeling approach, there are specific research questions that are better handled in a LVM framework.

## Latent variable models

LVM approaches that are used to evaluate change over time include latent growth curve models (LGCMs McArdle and Epstein, 1987; Muthén and Curran, 1997). In an LGCM, change is modeled as a function of time and is represented through the specification of latent (i.e., unobserved) variables referred to as *growth factors*. A latent intercept and a latent slope (i.e., the growth factors) are estimated based on the individual trajectories. Growth factors provide an estimate of the average trajectory, and individual variation around that trajectory, over time. Since the growth factors are estimated (i.e., latent), they are considered random effects. These model parameters can provide insight into average change and individual difference surrounding that change; model fit indices can also be obtained for LGCMs. While LGCMs can be a useful tool for stress researchers working with biomarkers, they have not appeared in the literature frequently.

Ram and Grimm (2007) provide a tutorial for LGCMs in the context of cortisol research in developmental inquiries, specifically for aging. However, LGCMs have not breached their way into premier neuroscience or health psychology journals, to evaluate change over time in cortisol and alpha-amylase. This could be due to several reasons. First, researchers may not be aware that LGCMs can be relevant to provide insight into their complex hypotheses. Second, the estimation of LGCMs requires the knowledge of specialty software programs. To compound this issue, the coding languages vary across software programs. Mastering these coding languages can be rather arduous and time-consuming. Furthermore, the distinction between LGCMs and other approaches, such as multilevel models and MRMs, is not well pronounced. Researchers may not be aware of when an LGCM is more appropriate to use compared to these other approaches.

Within the LVM framework, we can start to incorporate extensions that allow for a more complete version of the stress response to be modeled. For example, the models we present in the current manuscript (e.g., the LGCM) can be viewed as “base models.” In other words, each of these models can be expanded in a variety of ways to incorporate manifest or latent variables influencing various parts of the growth model. These variables can be incorporated as predictors, covariates, outcomes, or distal outcomes. In this case, the model can represent a larger, inclusive system of variables that work together to better capture the stress response and promote a deeper understanding of the biomarker fluctuations that are related to the stress response. True flexibility of research questions is possible within the LVM approach.

This family of statistical techniques is, of course, not a novel concept. These models have been explored in a variety of substantive and methodological inquiries for decades. However, these statistical tools are lacking in the stress-related biomarker literature and we feel that incorporating them can help to broaden the scope of questions being examined.

## Specific goals and intended audience

Given that the typical statistical approach in biomarker related research can only answer mean-difference-based questions, we felt it was important to highlight the use of a potentially richer and more flexible statistical modeling framework. Since there is no set standard of methodology within the field due to the vastly different data collection patterns that can be used, researchers may not be aware of the most advantageous and appropriate statistical tool for their situation. Our hope is that researchers examining the impact of the stress response on biomarker fluctuations will find these LGCMs useful when constructing future research questions to explore. Therefore, the goal of this paper is to present statistical methodology, in a user-friendly manner, which can answer research questions that are important and under-studied in the stress-related biomarker field. Not only do we discuss how to formulate and interpret findings from relevant LGCMs, but we also show how easy they are to implement by including sample code for a variety of models. This code is available in our online Appendix.

## Hypothetical example

The Trier Social Stress Test (TSST) is a method for inducing a psychological stressor and evaluating the effects on biological responses (Kirschbaum et al., 1993; Kudielka et al., 2007). Several studies have confirmed that the TSST reliably induces activation of the HPA axis (Kudielka et al., 2004) and the sympathetic adrenal medullary (SAM) system (Nater et al., 2005). The TSST consists of a public speaking task and a verbal arithmetic task. The total procedure takes between 11 and 15 min to complete. Biological measures of the stress response can be collected before and after the TSST at several time-points (Kirschbaum et al., 1993); for a thorough description of the TSST protocol, see Kirschbaum et al. (1993) or Kudielka et al. (2007).

Describing the different specifications of the LGCM in the context of the TSST allows for the nuances of the models to be described in a way that is relevant and familiar to stress researchers. The types of questions that can be answered, and the modeling issues that may arise, will then be described in the context of a commonly used experimental paradigm in stress research. However, this modeling approach can also be implemented in a variety of longitudinal research settings outside of stress research.

## Basic details surrounding latent growth curve models

LGCMs are a class of LVMs designed to capture change over time. Within the context of most biomarker research, data are skewed and need to be transformed before analysis (Miller and Plessow, 2013). However, there have been many advances for LVM techniques, and now any type of variable (e.g., those on different scales of metric) can be modeled without data transformation (Muthén and Asparouhov, 2002). If the item-type (e.g., binary, ordered categorical, or count) is properly specified in the code, then the software program selects the optimal estimator and data need not be transformed.

Another important factor to consider before estimating an LGCM is the number of time-points data were collected over. In order to test a linear trend, at least three time-points are needed (i.e., three points make a line). When model complexity increases, as when evaluating nonlinear change, the number of time-points needed also increases; for example, complex nonlinear change over time may require four, or more time-points (e.g., Grimm and Ram, 2009). LGCMs can also model time-points that are either equal or unequal regarding their spacing. For the basic LGCM, each subject is typically measured on the same measurement occasions (i.e., each subject shares the same time-points, but those time-points need not be equally spaced). However, basic LGCMs can be easily expanded to account for subjects on different measurement occasions (see Muthén, 1997 or Muthén and Muthén, 1998–2016 for further information and an example of implementation).

**Figure 1:**
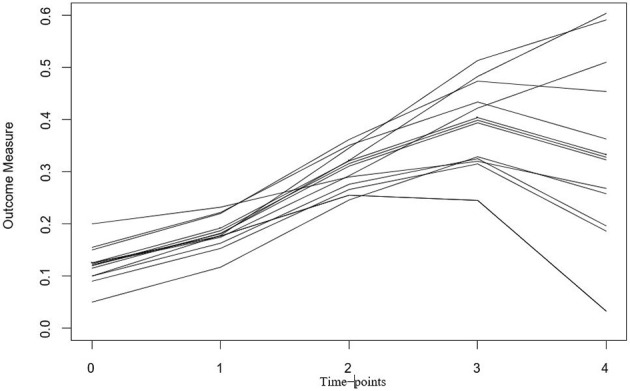
Example trajectory plot for a Latent Growth Curve Model (LGCM). Each line (or trajectory) represents an individual persons growth trajectory across time. In the case of the example, this could be how the stress response (i.e., outcome measure) changes over-time across longitudinal measurements of data.

LGCMs capture change over time through the specification of latent (i.e., unobserved) growth factors. Latent growth factors represent change through the estimation of a latent intercept (i.e., initial level) and latent slopes (i.e., rate of change), which can reflect linear or nonlinear growth patterns (Grimm and Ram, 2009). The intercept and slope are latent because they are not variables that exist in the data set. Rather, they are estimated based on the collection of trajectories obtained for each individual. Figure 1 is an example of a plot containing individual growth trajectories. The intercept is reflected by the *y*-axis, and the slope reflects the rate of change over the time-points (*x*-axis). Each line represents an individual's trajectory from which an intercept and slope(s) are estimated. These trajectories can then be summarized by an average growth trajectory and measures of variance surrounding the average trajectory. The measures of variance represent the individual slopes surrounding the average trajectory and provide insight into inter-individual differences within the overall growth pattern(s) captured.

## Sample size limitations of LGCMs

There are some important limitations that need to be considered when deciding between LGCMs and other methods of evaluating change over time. Possibly the most relevant limitation to stress researchers is the issue of sample size. LGCMs can require larger sample sizes than other approaches (e.g., MRMs), especially with fewer time-points or as model complexity increases; some studies suggest needing over 1,000 subjects, depending on model complexity (see Hertzog et al., 2006; Hertzog and von Oertzen, 2008; Cheong, 2011). However, studies have found that basic LGCMs can perform adequately under smaller sample size situations. For instance, Cheong (2011) found that LGCMs had adequate power to model mediation relationships when the sample size was 200 and there were at least five time-points. Furthermore, Fan (2003) found that, when evaluating linear growth, LGCMs had more power to detect group differences in latent trajectories than repeated measures ANOVA did. Regardless, sample size is an important limitation because the cost of collecting biomarkers can grow quickly as the sample size increases, especially if researchers do not have a wet-lab locally and have to ship out samples. If the research question dictates the use of LGCMs, and large sample sizes are not achievable, there are alternative methods that can be considered.

Bayesian estimation is an alternative modeling framework that allows researchers to incorporate subjective information into their statistical models that can have a similar effect as increasing power to detect effects (Kaplan and Depaoli, 2013). For example, Zhang et al. (2007) found that the LGCM could be estimated with as few as 20 subjects under the Bayesian estimation framework. A thorough review of Bayesian estimation and the specific issues involved are beyond the scope of this paper. For less technical reviews of Bayesian estimation, see van de Schoot and Depaoli (2014) and van de Schoot et al. (2014). For a more technical review of Bayesian estimation and how it applies to LVMs such as LGCMs, see Muthén and Asparouhov (2012) and Kaplan and Depaoli (2012).

## Unique benefits to biomarker research

The latent growth modeling framework encompasses many forms of LGCMs, which carry unique benefits in biomarker research. Perhaps the most pronounced benefit is that we see an extension of types of research questions that can be examined using this modeling framework. Of course, there are other statistical approaches that would also help to expand topics currently being explored beyond mean-difference-based inquiries. Such approaches include multilevel models and MRMs. There are many areas of overlap between these two modeling approaches and the LVM framework we discuss. Some obvious connections are the fact that latent growth models are indeed multilevel models and that mixed effects can be specified in all approaches. We view the LVM framework as just one approach that can help to broaden the scope of research questions being examined. One important extension that the LVM framework provides is the use of multiple indicators for a single construct. In other words, constructs can be included into any of these types of growth models as latent variables with many observed indicators (e.g., items on a scale). These latent constructs can be included as predictors, covariates, or (distal) outcomes within the “base” latent growth modeling being examined (see for e.g., Gunnell et al., 2016). This feature represents the extreme flexibility of the LVM framework. We cover additional benefits for biomarker research specific to each of the types of models presented in the following sections.

The following main section presents key specifications of the LGCM relevant to stress related inquiries using biomarkers. The organization of the remaining sections is as follows. First, a description of path diagrams and how they are used to represent LGCMs will be provided. Next, we present a sampling of the types of research questions that can be addressed using LGCMs. This is followed by a description of four specifications of the LGCM, as well as issues surrounding model fit. Each specification of the LGCM will be discussed in the context of the TSST and how they can address specific research questions. We focus on the basic LGCM, a multi-group version, the piecewise LGCM (PLGCM), and the LGCM for two parallel processes. We cover issues tied to assessment of model fit and adequacy related to these models. Finally, two statistical software packages to estimate LGCMs will be discussed; namely, M*plus* and the freely available program R.

## Path diagrams

Each of the specifications of the LGCM will be described in reference to a corresponding path diagram, which represents any type of LVM in a convenient graphical form. These diagrams allow LGCMs to be discussed through a graphical representation rather than through an equation. In a path diagram, squares or rectangles represent manifest or observed variables that would appear in the data file (e.g., cortisol). Circles represent latent or unobserved variables, such as the growth factors (e.g., latent intercept and slopes) in LGCM. Manifest and latent variables are linked together by paths with arrows at the ends. Single-headed arrows represent paths in which one variable predicts another variable (i.e., a regression path). Double-headed arrows represent the covariation or correlation between two variables, latent or manifest. Numbers beside the paths represent relationships between variables that are fixed in order to preserve the structure of the desired growth model being estimated. Paths without any numbers indicate relationships between variables that will be freely estimated.

## Types of important and under-studied research questions

The impact of the stress response on fluctuations in biomarkers is a broad topic, and we see room for improved flexibility in the research questions currently being addressed in the field. This section presents five types of research questions that can be answered using the specific LCGM techniques described below:

1. What is the continuous rate of change in cortisol?
2. What does the change in cortisol look like over time?
3. How do cortisol and alpha-amylase relate over time?
4. Are there (observed or unobserved) group differences in the rate of change in cortisol?
5. Does the rate of change in cortisol predict health outcomes?

These questions are a sample of the types of important and under-studied research questions that can be answered using LGCMs to evaluate change in biomarkers. This is not an exhaustive list of the types of research questions that can be addressed using LGCMs, nor is it a complete list of questions that can be addressed within biomarker research. Rather, the questions were selected to provide a context to discuss the implementation of the different LGCM specifications. Table 1 presents the five research questions in more detail. We present sections on several main specifications of the LGCM. Within each model-specific section, we describe the relevant research questions and how they can be addressed. Issues such as model fit and assessment, as well as issues related to statistical software, are also described.

**Table 1:** Types of questions each specification of the LGCM can address.

**Question**	**Basic LGCM**	**LGMM**	**PLGCM**	**LGCM for 2 parallel processes**
1. What is the continuous rate of change in cortisol?	Provides information on the rate of cortisol change throughout the study. When nonlinear slopes are specified, gives insight into the rate of the nonlinearity present.	Provides information on how the rate of change in cortisol differs across unobserved groups.	Provides information on the rate of cortisol change during the baseline phase (slope 1) and the recovery phase (slope 2)	Provides information on the rate of change for two processes (i.e., cortisol and alpha-amylase), whether linear or nonlinear
2. What does the change in cortisol look like over time	Can gain insight into the stress reaction and the recovery period through the specification of nonlinear growth factors.	Provides information on how the trend differs across unobserved groups (e.g., linear in one group and quadratic in another).	Can evaluate the change in cortisol over time for the baseline and recovery periods separately.	Can address in the same way as the basic LGCM, LGMM, and PLGCM depending on specification. Addresses these question for each process and provides insight into how they are related across processes.
3. How do cortisol and alpha-amylase relate over time?	Can evaluate growth of cortisol and alpha-amylase through a multivariate LGCM or can control for the effect of alpha-amylase at each measurement of cortisol.	Provides information into how these relationships differ across unobserved groups.	Can evaluate growth of cortisol and alpha-amylase through a multivariate PLGCM or can control for the effect of alpha-amylase at each measurement of cortisol.	Evaluates how activation of each system is related through relationships specified between growth factors of each system.
4. Are there (observed or unobserved) group differences in the rate of change in cortisol?	Can control for the effects of a grouping variable (i.e., time-invariant covariate) or can compare the trajectories and rates of changes of each group.	Can evaluate differences in the trajectories and rate of changes for unobserved groups (e.g., extreme responders vs. normal responders)	Can control for the effects of a grouping variable (e.g., gender) or can compare the trajectories and rates of changes of each group.	Can control for the effects of a grouping variable (e.g., gender) or can compare the trajectories and rates of changes of each group.
5. Does the rate of change in cortisol predict health outcomes?	Can answer whether the rate of change in cortisol affects a health outcome (e.g., the number of medical office visits)	Provides insight into how cortisol predicts health outcomes may differ across unobserved groups	Can answer whether the rate of change in cortisol at baseline or recovery affects a health outcome (e.g., the number of medical office visits).	Same as the basic LGCM and the PLGCM, except can now include how the rate of change in alpha-amylase also affects health outcomes (e.g., the number of medical office visits).

*LGCM, Latent growth curve model; PLGCM, piecewise LGCM; LGMM, latent growth mixture model*.

## The basic LGCM

**Figure 2:**
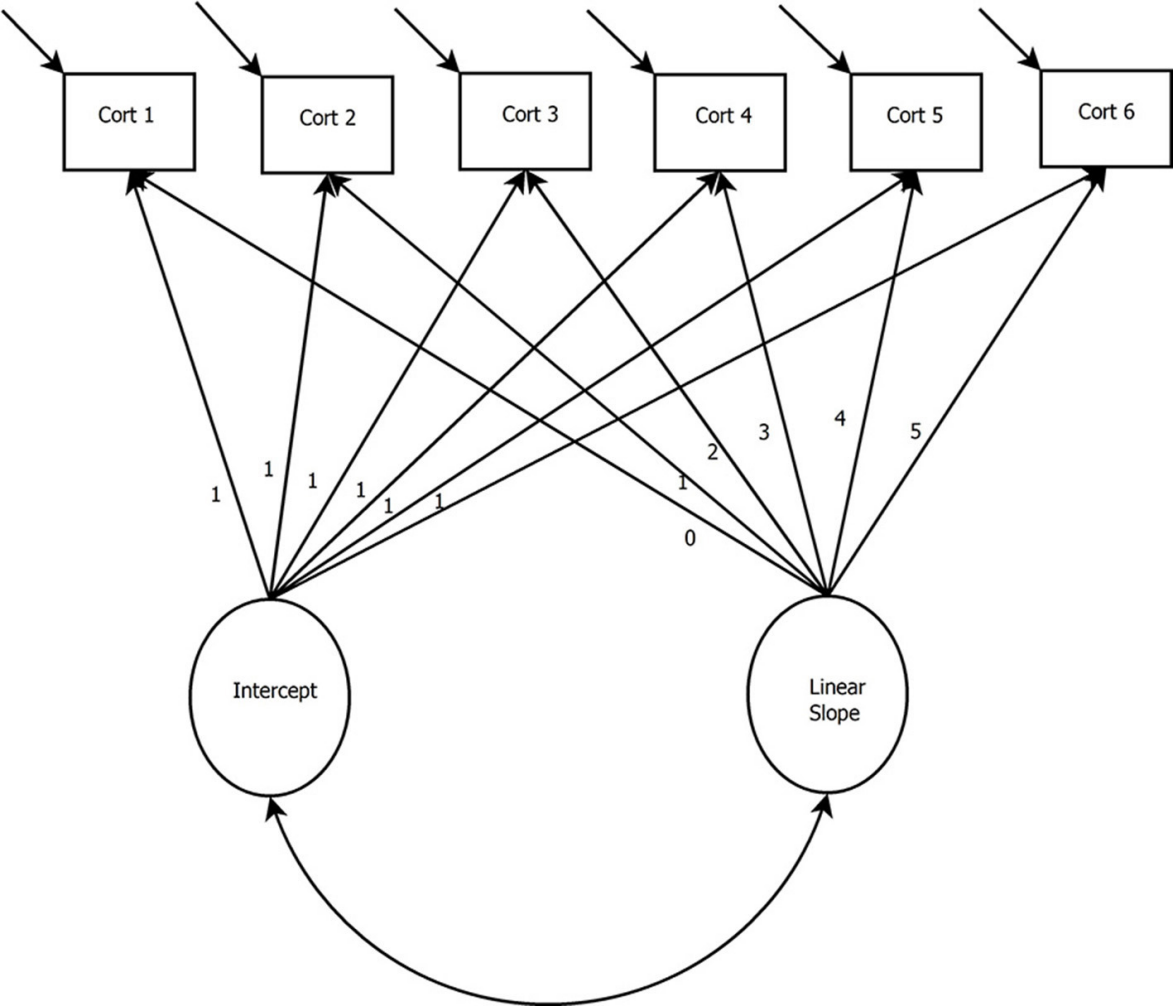
Latent Growth Curve Model with a Linear Slope. *Cort, Cortisol measurement occasion*.

Figure 2 presents a path diagram of a basic LGCM. The basic LGCM was developed to evaluate the continuous rate of change over time (McArdle and Epstein, 1987). In the basic LGCM, a latent intercept and a latent linear slope is estimated to capture linear change over time. This model is appropriate when the researcher does not expect bends in the trajectories over time.

An example of the basic LGCM can be found in Hagger-Johnson et al. (2010) who aimed to investigate the effects of chronic stress on physical and mental well-being. The linear slope from a basic LGCM yielded a significant relationship between the rate of change in cortisol levels to mental health issues. Specifically, a steeper decline in daytime cortisol levels related to better mental health scores, indicating a link between chronic stress and mental health. However, results from this study are cross-sectional and causation could not be determined. In experimental designs, nonlinear growth factors may need to be estimated to capture the bends in trajectories that an experimental paradigm may cause.

Burant (2016) investigates how LGCMs can be used to capture how depression levels change over time in elderly hospital patients. Using a combination of model fit indices and parameter estimates that correspond with theory, Burant (2016) determined that an LGCM with freely estimated slopes (akin to the latent basis model described below) best described changes in depression over time. This specification of the LGCM was able to capture nonlinear change and provide insight into where the greatest rate of change in depression occurred. Specifically, depression levels declined most quickly after 1 month, with the rate of change slowing until it reached its lowest levels at 6 months. One caveat to this model is that it requires larger sample sizes due to more parameters being estimated. For the description of the basic LGCM in this manuscript, quadratic growth will be the focus because of the context in which the models are described (i.e., TSST).”

**Figure 3:**
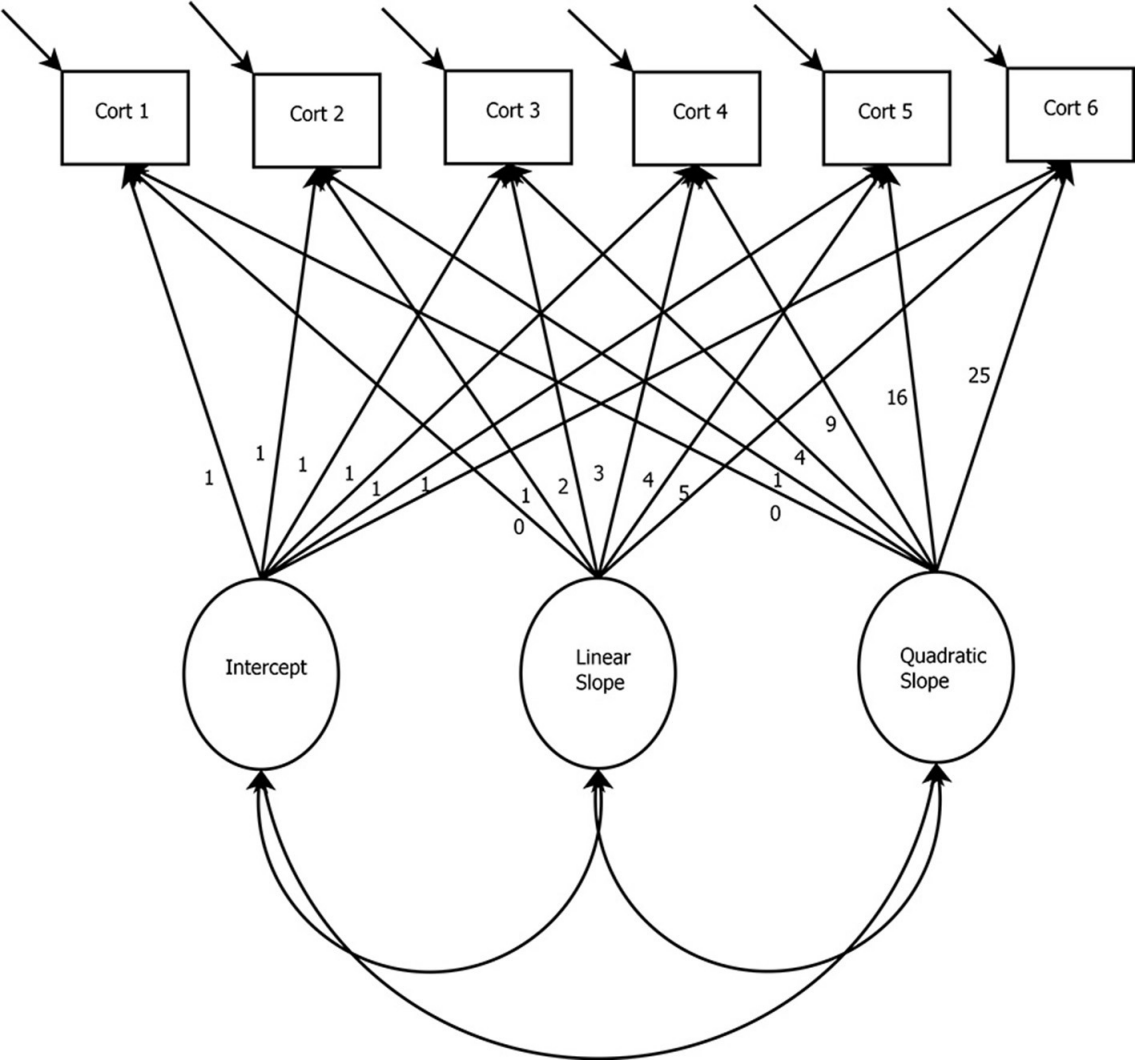
Latent Growth Curve Model with Linear and Quadratic Slopes. *Cort, Cortisol measurement occasion*.

In the model presented in Figure 3, a basic LGCM with a quadratic growth factor and six measurement occasions of cortisol is depicted. The latent variables in this model are the intercept and slope (i.e., linear and quadratic) growth factors. When interested in capturing the increase and decrease of biomarkers, nonlinear slopes can be specified (e.g., the quadratic slope in Figure 2). For the growth factors to represent change over time, the paths between the cortisol measurement occasions and the growth factors may be fixed to specific values. For example, Figure 2 shows the paths from the linear slope term fixed to the following: 0, 1, 2, … 5. Fixing these paths is a way of specifying a particular growth shape within the model (e.g., linear or quadratic). However, for extensions of the LGCM, such as the latent basis model, these loadings can be freely estimated to model any form of nonlinearity. In the case of Figure 2, these fixed paths from the slope terms would be freed and estimated to represent the degree of nonlinearity in the data (i.e., the paths would no longer say: 0, 1, 2, … 5).

In the basic LGCM, the intercept is specified by fixing all paths between the cortisol measurements and the intercept growth factor to one. The linear slope is defined by fixing the path between the first measurement of cortisol and the linear slope growth factor to zero. This specification makes the intercept represent the first time-point. However, any time-point can be specified to represent the intercept. Paths between subsequent cortisol measurements and the slope growth factor are fixed to represent equal spacing (i.e., with unit increments, as in Figure 2) or unequal time spacing (i.e., 0, 1, 4, 5 would indicate longer time has elapsed between the 2nd and 3rd time-points). The quadratic slope is specified through the squared values of the linear slope. Quadratic growth is only one form of nonlinear change, and higher order forms can be specified if desired (see e.g., Grimm and Ram, 2009).

## Unique benefits of the basic LGCM to biomarker research

As we will illustrate in subsequent sections, the LGCM is a highly flexible model that can be manipulated in a variety of ways to answer complex and dynamic research questions. Arguably, one of the most beneficial modifications that can be made to the basic LGCM is to specify various forms of nonlinear change within the model. The LGCM can be estimated in a variety of ways, each capturing a different picture of what kind of growth patterns exist in the data. The LGCM can capture nonlinear change through the specification of polynomial growth factors (e.g., quadratic or cubic), a feature that it shares with MRMs (e.g., quadratic and cubic; Meredith and Tisak, 1990; Willett and Sayer, 1994; Bauer, 2007).

However, LGCMs carry added flexibility and can handle other forms of nonlinearity, which often cannot (easily) be implemented in other modeling frameworks (e.g., using MRMs). For instance, alternative specifications of the LGCM exist where the pattern of nonlinear change can be estimated. The *latent basis model* is one such model that treats the pattern of change as a latent variable (McArdle and Epstein, 1987; Meredith and Tisak, 1990). In this type of model, the user need not implement a pre-specified growth pattern (e.g., quadratic growth). Instead, the pattern of change is estimated as latent.

Another form of handling nonlinearity is to use an additive model (e.g., the generalized additive model; Hastie and Tibshirani, 1986), which is used to identify nonlinearity without specific knowledge of where the bends in the trajectory (i.e., changes in growth patterns) are located. Example code for these two forms of handling nonlinearity in the model is included in the online Appendix. There is a wide range of modeling techniques that can be used to incorporate or assess nonlinear change within LGCMs, and these are just two examples. For more information on some of these, see Grimm and Ram (2009), Grimm et al. (2011), or Ram and Grimm (2007). For models that are nonlinear in the parameters (i.e., with binary indicators), see Blozis and Harring (2016). Due to the flexibility of the LVM framework, these assessments of nonlinearity can also be incorporated into the more complex versions of the LGCM that are discussed below.

Another benefit of evaluating growth in the LVM framework is the flexibility of the outcomes that can be handled. While both MRMs and LGCMs can model multivariate growth, LGCMs have a little more flexibility in how multiple variables are modeled. First, LGCMs can be used to evaluate the growth of other latent variables (Muthén and Asparouhov, 2002; Cheong et al., 2003). This is useful when a construct under study has multiple indicators (e.g., multiple measures of the SAM system). This modeling framework can also be used to handle autoregression, when outcomes at different time-points are allowed to predict one another (e.g., time 1 score predicts the score at time 2); see Bollen and Curran (2004) for more information. The addition of autoregressive elements in the models may be particularly relevant to the stress response because outcome measures are inherently related to measures collected at previous times within person. These are merely included to act as examples of the flexibility of the general LVM framework. Our main focus here is on the LGCM and it's immediate extensions into biomarker research.

## Research questions related to the basic LGCM

Table 1 presents several types of important and under-studied research questions. Specifically related to the basic LGCM, we can highlight Questions 1, 2, and 5 for this discussion. Question 1 examines whether the rate of change in a given outcome (e.g., cortisol) is continuous in nature. Fitting an LGCM to the data allows the researcher to examine this continuous rate of change and explore different growth patterns, which is closely tied to the next type of research question. Question 2 relates to what patterns of change look like over time for a given outcome (e.g., cortisol). The basic LGCM allows us to examine continuous change over time and express different forms of nonlinearity within the model to uncover the best model that captures patterns of change in the data. Finally, Question 5 examines how the rate of change in a repeated measures outcome (e.g., cortisol) might impact another outcome measure (e.g., other health outcomes). This specification can yield insight into, for example, how changes in cortisol caused by the TSST affects the number of medical visits. The LGCM can be easily modified to act as a predictor model for other outcomes, which can be measured at a single or multiple time-points. For full details on how the basic LGCM can be specified to address a range of questions, see Table 1.

## Multi-group (observed or unobserved) growth model

The basic LGCM can be extended to handle multiple groups. In this case, the researcher may be interested in examining growth or change-rate differences across different groups of individuals. These groups can be observed groups such as gender, race/ethnicity, disease status, or age. In this case, the model would be called a *multi-group LGCM*, which indicates that the groups are observed. However, groups can also be unobserved, or latent. In this case, the theory is that the sample data were collected for multiple unobserved subpopulations, where individuals from these populations follow different growth patterns. This type of model is often referred to as a *latent growth mixture model* (LGMM), where the word *mixture* indicates that groups are unobserved (i.e., the grouping label for an individual is something that is estimated in the model and is not a label that appears in the data file). Examples of latent groups can include individuals representing different levels of addiction status (Muthén and Shedden, 1999), individuals experiencing different outcomes of a traumatic experience (deRoon-Cassini et al., 2010), and adolescents with different smoking behaviors (Colder et al., 2001). In the context of the current paper, we might consider a subset of subjects that have a faster recovery time from the TSST compared to another group of subjects. In this case, the LGMM can be used to identify and model these subjects.

For the purposes of discussion, we will continue describing the LGMM here, but the (observed) multi-group LGCM would look much the same (code for both is provided in the online Appendix). When comparing observed multiple-groups to one another, there is typically an iterative process implemented. In particular, constraints are placed within the model (e.g., on the growth factor loadings if freely estimated, the parameter variances, or the covariances) one-by-one to assess exactly where (if at all) the model results differ across groups. This process helps to uncover how growth processes may differ across the observed groups. For more information on this process, please see Li et al. (2001) for an applied example or Muthén and Curran (1997) for a more technical description. The unobserved groups are (typically) handled in a different manner.

**Figure 4:**
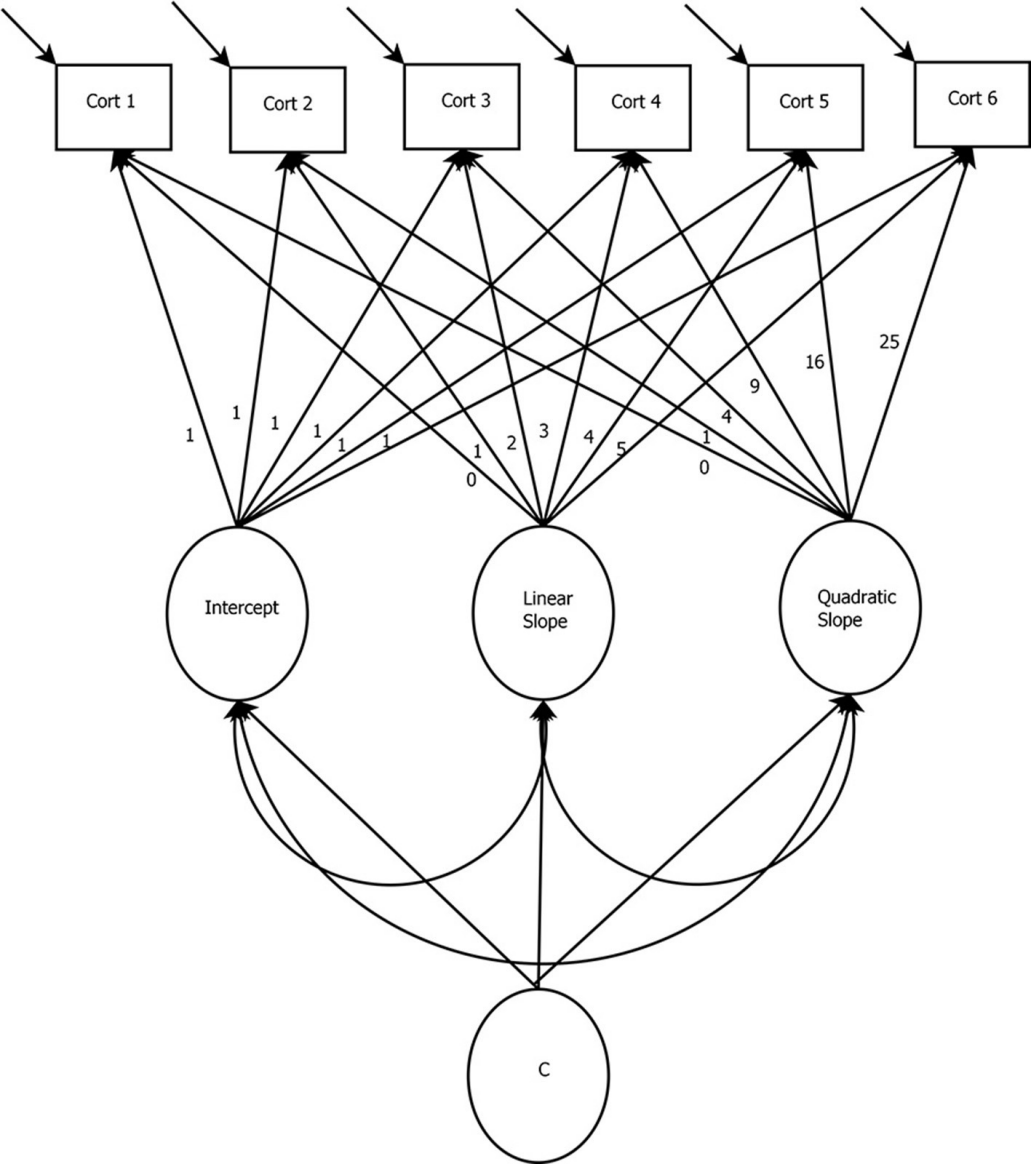
Latent Growth Mixture Model: In this specification, there is a linear and a quadratic trend estimated, but the relationships can differ across latent groups (c). Note that groups can also be observed (e.g., gender) rather than latent.

**Figure 5:**
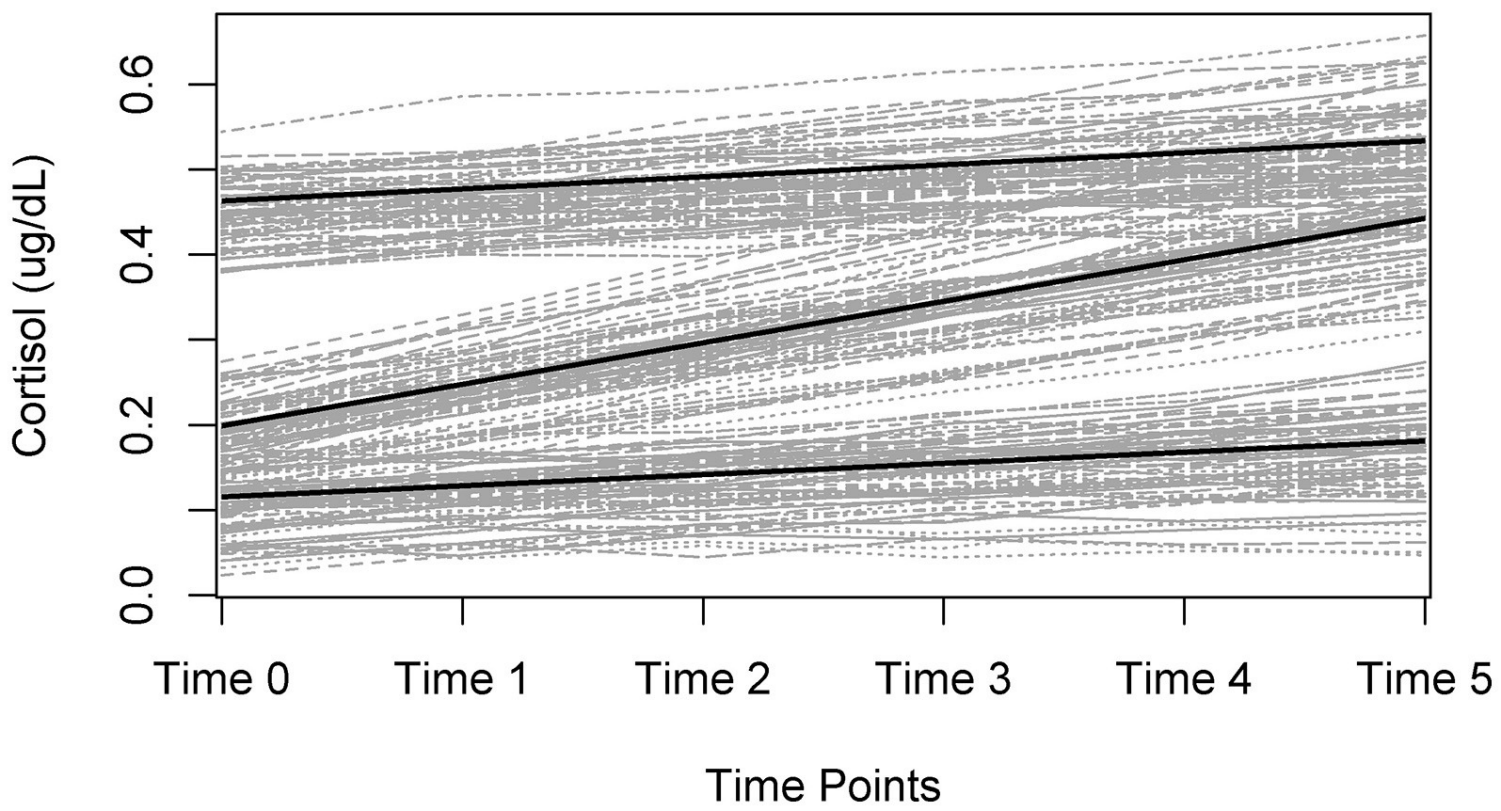
Trajectories for multiple groups (observed or unobserved).

As mentioned, the LGMM differs from the basic LGCM only in that multiple unobserved groups (or *latent classes*) are accounted for in the model. The user would estimate the model many times, each with a different number of latent classes specified. Then model fit assessments (described below) and substantive knowledge would be used in combination to determine the “best” number of latent classes, each represented by a substantively different growth trajectory. In other words, the LGMM identifies subpopulations that may have been sampled and estimates an LGCM for each unobserved group that has been identified. It is important to note that LGMMs are highly complex and should be estimated using a set of guidelines. Such guidelines have recently been published in van de Schoot et al. (2017). Some further modeling concerns about assumptions that have to be made in LGMMs can be found in Bauer (2007). A depiction of the LGMM can be found in Figure 4. Notice that the only difference between this model and the basic LGCM is the inclusion of the latent variable “c,” which indicates that the entire model is allowed to be estimated for latent groups such that each group can be represented by its own estimated growth trajectory. An LGMM trajectory plot might look something like Figure 5, where there are groups of trajectories that represent different growth patterns. In this case, we might identify three groups, each with their own estimated growth trajectory.

## Unique benefits of the LGMM to biomarker research

The multi-group approach to the LGCM is incredibly helpful for modeling different sub-groups on the same outcomes, and then doing subsequent comparisons across those groups. The LVM framework allows for these groups to be either observed or unobserved in nature, with the latter being a specific benefit to working within this modeling context. The ability to model latent groups allows researchers to explore potentially substantively interesting sub-populations and related covariates. This feature could be particularly beneficial when examining whether patterns of change are dictated by underlying characteristics that have not been previously explored.

## Research questions related to the multi-group growth model

The type of research question listed in Table 1 that is particularly relevant to this type of latent growth model is Question 4. This question can be used to explore whether there are viable groupings of individuals that substantively differ in their growth rates. When using the TSST, the LGMM may be able to distinguish between high and low responders to the TSST and estimate trajectories for each group. This provides insight into what the stress response of these two different types of responders looks like. The model can also be used in the context of large-scale models, which include additional covariates, and outcome measures. Perhaps the groups appear similar in their growth patterns, but differ substantively on other aspects of the larger model—this sort of model can help the researcher to distinguish these nuances. Any of the other research questions listed in Table 1 could be potentially relevant to this group of models, but Question 4 is the research question unique to this type of model. For a nice example showing how the LGMM applies to diurnal cortisol data, see Dmitrieva et al. (2013) or Ram and Grimm (2009).

## Piecewise LGCM

**Figure 6:**
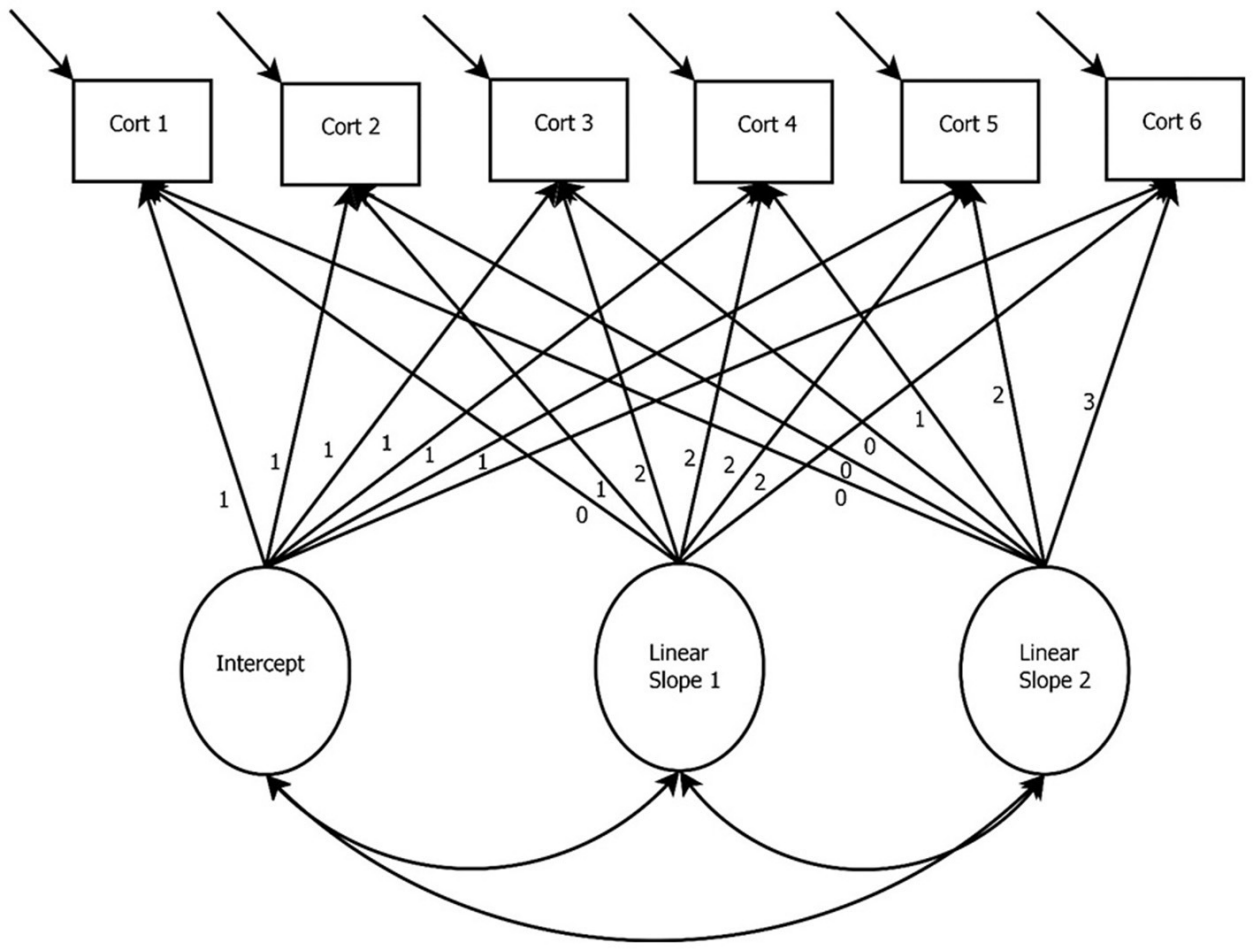
Piecewise Latent Growth Curve Model. In this specification, there are two phases being modeled, Phase 1 (cort1, cort2, and cort3) and Phase 2 (cort 4, cort 5, and cort 6). This relationship is defined through the slope paths. Phase 1 represents the time-points before the onset of the TSST (i.e., the baseline period), whereas Phase 2 represents the time-points after the onset of the TSST (i.e., the recovery period). *Cort, Cortisol measurement occasion*.

The PLGCM is also known as the multiphase LGCM or the spline LGCM. Figure 6 presents a path diagram of a two-piece PLGCM. In this specification, there are two phases being modeled, Phase 1 (cort1, cort2, and cort3) and Phase 2 (cort 4, cort 5, and cort 6). Phase 1 represents the time-points before the onset of the TSST (i.e., the baseline period), whereas Phase 2 represents the time-points after the onset of the TSST (i.e., the recovery period). The time- points for Phase 1 and Phase 2 will differ depending on how the study was designed (e.g., more time-points in the recovery period). Additional “pieces” can be specified in the model when the location of more than one bend is known (e.g., a baseline period, a reaction period, and a recovery period). The first piece (i.e., growth factor slope 1) represents linear change in cortisol before the onset of the TSST. The second piece (i.e., growth factor slope 2) represents linear change in cortisol after the onset of the TSST. For this example, we included three waves of data for each phase. The purpose of this was to ensure that each phase would be identified on its own (see Bollen and Curran, 2006). It is possible for each phase to only have two waves, akin to a confirmatory factor analysis with correlated factors and two indicators each. However, Diallo and Morin (2015) found that LGCMs with only two indicators may be underpowered to detect an effect. Therefore, discussion of this model focuses on three waves for each phase of the PLGCM.

Attention to the specifications of the paths between the observed items and the latent growth factors is crucial for estimation of the PLGCM. In Figure 6, the linear growth factor for the first phase is specified with the first three timepoints (i.e., cort1, cort2, and cort3) fixed to “0,” “1,” and “2,” respectively. Specifying the first three timepoints this way permits the estimation of the linear slope (similar to basic LGCM). However, the final three timepoints are fixed to “2.” This specification prevents information from the final wave of timepoints from being included in the estimation of the first linear slope. In other words, fixing the remaining timepoints to “2” allows the remaining growth information to be absorbed into the second piece. This generalizes to the second linear slope where the timepoints from the first three waves (i.e., cort1, cort2, and cort3) are fixed to “0.” This specification prevents information from the first three waves from being absorbed into the estimation of the second growth factor (Diallo and Morin, 2015). This allows each piece to capture information across different phases of the trajectory.

## Unique benefits of the PLGCM to biomarker research

The PLGCM is an alternative specification of the LGCM for researchers interested in capturing nonlinear change over time when there is knowledge as to the location of the bend in the trajectory (Kohli and Harring, 2013). The PLGCM is specifically relevant to the data collection protocol using the TSST since growth rates can be viewed as different phases—before and after the acute stressor.

## Research questions related to the PLGCM

Research Questions 1, 2, and 5 (see Table 1) are most relevant to this type of growth model. Akin to the basic LGCM discussed above, Questions 1 and 2 can also be addressed through the piecewise version of this model. Given that the location of the bend in the trajectory will be known when implementing the TSST, the PLGCM can provide more accurate insight into the rate and nonlinear change in cortisol. With the PLGCM, Question 1 can be addressed in two parts: rate of change in the reactivity period (first linear slope growth factor), and rate of change in the recovery period (second linear slope growth factor). Question 2 is addressed because the location of the bend in the trajectory is specified (rather than estimated) and can provide more accurate results regarding the growth trajectory. The PLGCM can also be easily extended to address Question 5 when assessing whether the piecewise growth curve acts as a predictor for any other health outcome. This notion falls into the inherent flexibility of the LVM framework.

## LGCM for two parallel processes

**Figure 7:**
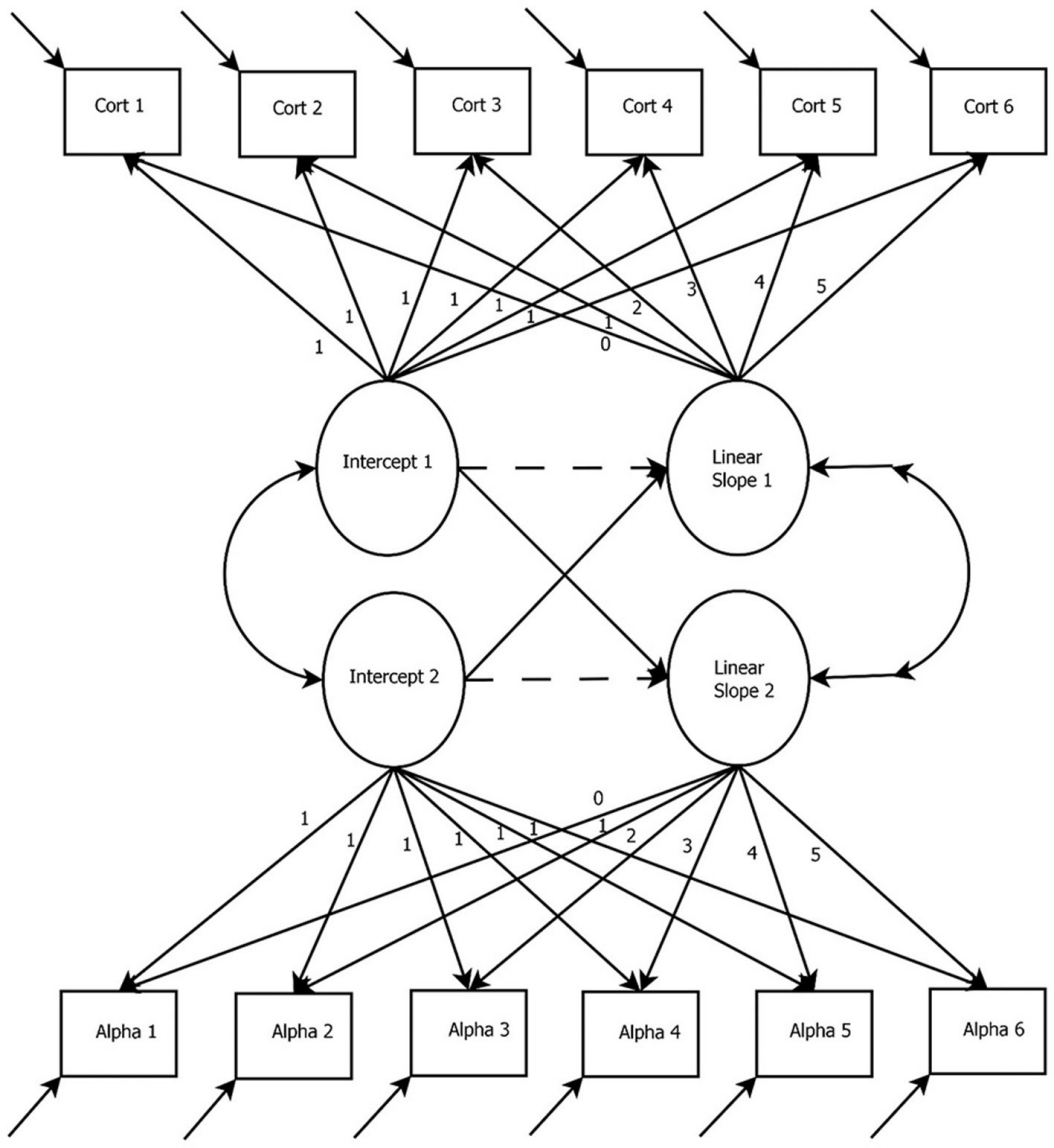
Latent Growth Curve Modeling for Two Parallel Processes. The intercept and slope terms can be related in a variety of ways. For example, Intercept 1 can predict only Slope 1, only Slope 2, or both slope terms. Dashed lines have been included from the corresponding intercept and slope terms to show the choice of including this relationship or not within the model being estimated. *Cort, Cortisol measurement occasion; Alpha, Alpha-amylase measurement occasion*.

Figure 7 presents a path diagram for the LGCM for two parallel processes [also referred to in the literature as the Bi-variate LGCM (Muniz-Terrera et al., 2017), multivariate LGCM Bollen and Curran, 2006, multiple domain LGCM (Byrne and Crombie, 2003), and the associative LGCM Bollen and Curran, 2006]. This model is an alternative specification of the LGCM for researchers specifically interested in how the trajectories of two systems are related to one another (Cheong et al., 2003). In this example, trajectories for cortisol and alpha-amylase are simultaneously estimated through separate growth factors. In other words, there are two cortisol-specific growth factors (i.e., intercept and slope) and two alpha-amylase-specific growth factors estimated. The way that the trajectories of cortisol and alpha-amylase relate can now be modeled through the relationships of their specific growth factors. Figure 7 provides an example of how the relationships between the growth factors of these two biomarkers can be modeled. The direct paths between the latent growth factors of cortisol and alpha-amylase can be specified in any way to accommodate specific research questions about how these processes relate. Most importantly, the specification of the LGCM for two parallel processes presented in Figure 7 is specified to estimate linear change of both processes. Because there will be nonlinear change in both cortisol and alpha-amylase due to the TSST, the LGCM for two parallel processes can be specified to account for nonlinear change. The LGCM for two parallel processes can also be specified to account for nonlinear growth change (e.g., quadratic or freely estimated slopes), or it can be combined with the PLGCM when the location of the bend in the trajectory is known. Ultimately, decisions for specifying the LGCM for two parallel processes should be driven by the research questions and data characteristics.

## Unique benefits of the parallel process LGCM to biomarker research

The parallel process model is perhaps the most encompassing model that we describe here. This model shows potential to be most complex because any of the other LGCM variations we have discussed can be embedded within each process within this model (e.g., one process can include a mixture component, piecewise growth, etc.) The benefit of this model is that it allows researchers to be extremely malleable when research questions are being developed. There need not be a single outcome measure across time, and other elements (e.g., mixture components and nonlinear growth curve functions) can be embedded in different ways within each of the processes.

## Research questions related to the parallel process LGCM

**Figure 8:**
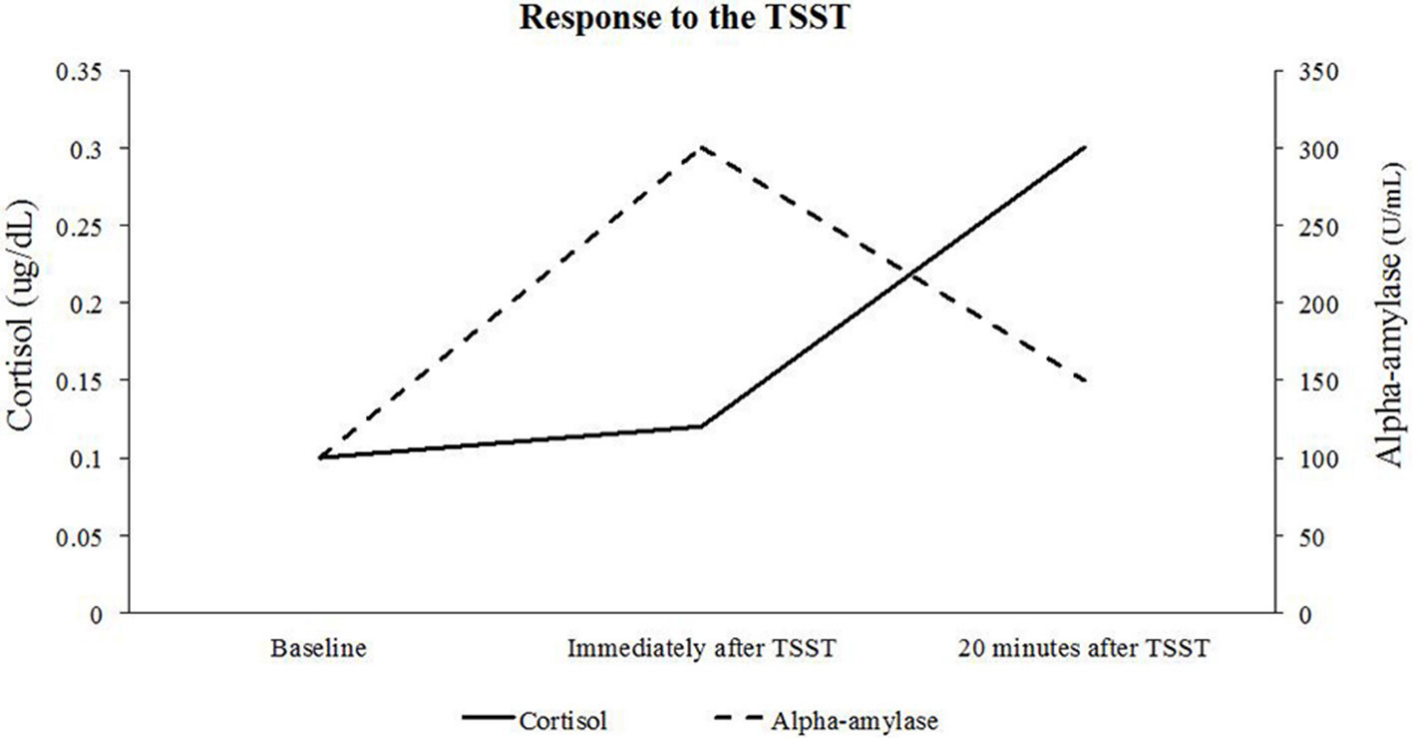
Trajectories for cortisol and alpha-amylase during the TSST. This figure shows that changes in alpha-amylase occur immediately after the stressor, whereas changes in cortisol occur about 20 min after the stressor.

Research Question 3 (see Table 1) is most applicable to the parallel process LGCM. This question deals with how two separate outcomes can relate over time. However, it is also important to note that the LGCM for two parallel processes can answer any of the other questions in Table 1 for each process simultaneously. The relationship between cortisol and alpha-amylase is explicitly modeled in how the growth factors for each process are related. Specifically, the LGCM for two parallel processes provides insight into how the baselines (i.e., intercept growth factors) and rates of change (i.e., slope growth factor[s]) are related in each system. Modeling how the growth factors of cortisol and alpha-amylase are related can provide more insight into how activation of the HPA-axis is related to activation of SAM system. For full details on how the LGCM for two parallel processes can be specified to address a range of questions, see Table 1. Finally, for an example of plotting two parallel process growth trajectories, see Figure 8. In this figure, we can see that cortisol and alpha-amylase substantially vary in their growth patterns over time, even though the baseline assessment is comparable.

## Model fit and assessment

Model fit and assessment is typically an important part of implementing any type of LGCM. Model fit statistics are measures of how well a statistical model reflects the data. Model fit can be evaluated through two different classes of statistics: (1) absolute model fit, and (2) relative model assessment measures. The following sections will discuss the two classes of statistics and the types of questions that they can aid in answering when implementing LGCMs.

## Absolute model fit

Absolute fit statistics are used to determine how well a statistical model reflects the data. This category includes the closeness-of-fit measures, badness-of-fit measures, and the χ^2^ goodness-of-fit test (Bentler and Bonett, 1980; Bentler, 1990). Closeness-of-fit measures include the comparative fit index (CFI) and the Tucker-Lewis index (TLI); there are many additional measures, but these are arguably the most common because they are reported in most LVM software. CFI and TLI values close to 1.0 indicate a statistical model that adequately reflects the data. Generally, CFI and TLI values above 0.96 (for CFI) or 0.95 (for TLI) reflect excellent fit and values of 0.90 reflect mediocre fit (Bentler, 1990; Byrne, 1994; Schumacker and Lomax, 2004). Badness-of-fit measures include the root mean square error of approximation (RMSEA) and the standardized root mean square (SRMS). RMSEA and SRMS values closer to zero indicate a statistical model that adequately reflects the data. Generally, RMSEA, and SRMS values of 0.01 reflect excellent fit, values of 0.05 reflect good fit, and values of 0.08 reflect mediocre fit (MacCallum et al., 1996). The χ^2^ goodness-of-fit statistic indicates that a statistical model adequately reflects the data when the corresponding *p*-value is above the nominal 0.05 level. Caution should be exercised when interpreting the χ^2^ goodness-of-fit statistic as the statistic is sensitive to sample size, with larger sample sizes sometimes erroneously indicating model misfit (Satorra and Saris, 1985). These measures can all be used to assess whether a model fits the data or not. Sometimes there are inconsistencies in the results, where some measures indicate the model fits the data and other measures do not. If this discrepant result occurs, it is imperative to reflect on the substantive information driving the model when assessing the final model. It should be noted that model fit indices may not perform equally across all models and all modeling contexts, and reliance on the rule-of-thumb cut-offs can yield misleading results (Barrett, 2007; Hayduk et al., 2007; Nylund et al., 2007). For instance, Nylund et al. (2007) found that the correct number of mixtures in a growth mixture model were only identified by the bootstrap loglikelihood ratio test and the Bayesian information criterion (BIC) With this in mind, model selection and evaluation should not rely solely on these rules-of-thumb for absolute model fit. Rather, model evaluation should come from a combination of relative and absolute model fit, as well as how well the parameter estimates fit with previous literature or theory.

## Relative model assessments

Relative model assessment indices are used to compare competing statistical models and include information criteria (IC) and likelihood-ratio tests. IC assessments include the Akaike information criterion (AIC; Akaike, 1981), the Bayesian information criterion (BIC; Schwarz, 1978), and the sample-sized adjusted BIC (saBIC), to name a few. IC values can be compared across two or more models, where the first represents the original model and the subsequent models represent competing models (varying to some degree from the original model). The statistical model with the lowest IC is then selected as the optimal model; i.e., the one reflecting the data patterns best. Likelihood-ratio tests can also be used to compare two models via χ^2^-difference test. The difference in χ^2^ values between two models is calculated and compared to a χ^2^ distribution with degrees of freedom equal to the difference in parameters estimated between the two models. A *p*-value lower than the nominal 0.05 indicates that the models are significantly different from one another, and the model with the lower χ^2^ value is then selected as the optimal model. Relative model assessment measures are especially useful when researchers are trying to determine whether to assess linear or nonlinear change in the LGCM. Researchers can estimate one LGCM that specifies a linear slope and another LGCM that specifies nonlinearity. Then the model assessment measures can be used to help the researcher determine the pattern of change that best reflects the data patterns being modeled.

## Statistical programs to estimate LGCMs

The estimation of LGCMs requires the use of statistical software programs capable of estimating LVMs. There are several statistical software packages used to estimate LGCMs that differ in modeling capabilities, complexity of the program, and price. Table 2 presents the modeling features of the most commonly used statistical software programs for estimating LGCMs (i.e., Amos, SAS, Stata, Mplus, EQS, and Lavaan through the R programming environment). The purpose of Table 2 is to provide the reader with relevant features of each program so that they can make a decision as to which program best meets their needs. We discuss and provide code for three different software programs: M*plus* (Muthén and Muthén, 1998–2016), Amos (Arbuckle, 2014), and the R package Lavaan (Rosseel, 2012; R Core Team, 2015), with the latter being of no cost for users.

**Table 2:** Statistical methods for assessing growth and the software that can estimate these models.

**Method**		**SPSS: AMOS v. 23**	**SAS PROC CALIS v. 9.4**	**STATA v. 14**	**Mplus v. 7.4**	**EQS v. 6.3**	R: Lavaan^**^ v. 0.5-20
Latent Growth Curve Model		X	X	X	X	X	X
Latent Growth Mixture Model		X	X	X	X		
**Data Types**							
	Continuous	X	X	X	X	X	X
	Binary	X	X		X	X	X
	Ordered-Categorical	X	X		X	X	X
	Unordered-Categorical				X		
	Count			X	X		X
**Estimator**							
	ML/MLR	X	X	X	X	X	X
	WLS/WLSM/WLSMV/ADF		X	X	X		X
	Bayesian	X	X	X	X		X
**Missing Data**							
	Full-Information Maximum Likelihood	X		X	X	X	X
	Multiple Imputation	X	X	X	X	X	
Price of Program (In USD)^*^							
	Annual Academic License	811	9,200	445	175	NA	Free
	Perpetual Academic License	1840	NA	895	1095	595	Free

* *All prices are for the full versions of each program as of June 2016. In some instances, removing some features can reduce the cost of the program*.

** *R package Blavaan is a Bayesian extension of the R package Lavaan*.

M*plus* is one of the more flexible LVM programs. Researchers can evaluate change over time for any type of variable (i.e., continuous, binary, ordered-categorical, unordered-categorical, count, and censored). Furthermore, M*plus* has multiple options for handling missing data, including full-information maximum likelihood and multiple imputation. Compared to the other programs presented in Table 2, M*plus* is decidedly the most user-friendly with a website (http://www.statmodel.com) that contains example code for many different types of models. M*plus* is also capable of more advanced modeling techniques such as estimating unobserved groups (i.e., latent mixture modeling) and Bayesian estimation. The cost of this program is $1095 for an academic license, with an annual fee of $175 to keep the license current and qualify for upgrade downloads when made available. Example code and contrived data to estimate each of the LGCM specifications in M*plus* can be found in the online Appendix.

The next software package discussed is Amos (Arbuckle, 2014). Amos is another user-friendly program that has many of the same modeling capabilities as M*plus*. Rather than relying on a syntax-based coding language like M*plus* (and the R package, Lavaan, detailed next), Amos uses a graphical interface where the user specifies the model by drawing a path model using point-and-click tools. One limitation of Amos is that it is a more expensive program than other programs such as M*plus*, with an annual fee starting at $811.00 per license. Another limitation of Amos is that the modeling capabilities are less flexible than M*plus*, where features such as multilevel modeling can be combined easily with LGCMs. However, data management in Amos can still be housed in SPSS file formats, which may be easier to manage than text and csv files required by M*plus*. In order to estimate the specifications of the LGCM discussed, a user is able to draw the models as they appear in Figures 2–7.

The final software package discussed is the R package, Lavaan. Lavaan has many of the same modeling capabilities as M*plus*, but is a free program and provides the additional benefits of being part of the R programming environment (i.e., it is an open source program that is easily linked to other packages in R). One limitation of Lavaan is that it does not feature all of the modeling capabilities of M*plus*, such as multiple imputation or Bayesian estimation. However, there are R packages designed to handle these issues that can be used alongside (or instead of) Lavaan, such as the R package MI (Su et al., 2011) for multiple imputation and BLavaan (Merkle and Rosseel, 2016) for Bayesian estimation. While Lavaan is decidedly less user-friendly than other programs presented in Table 2, it is free and contains most of the modeling features of its more expensive counterparts. Example code and a contrived data set to estimate each of the specifications of the LGCM in Lavaan can be can be found in the online Appendix.

## Discussion

The aim of the current paper was to present LGCMs, in a user-friendly manner, which can answer research questions that are important and under-studied in the stress-related biomarker field. We discussed how to formulate and interpret findings from relevant LGCMs, and showed how easy they are to implement by including sample code for a variety of models.

LGCM methodology provides advantages to researchers interested in studying change over time of biomarkers of the stress response. The LGCM provides insight into the rate at which a variable changes over time through the specification of latent growth factors. Latent growth factors answer questions about the rate of change of a variable, and how that rate of change relates to other variables. Different specifications of the LGCM were selected to address specific research questions developed through the context of the TSST; namely, the basic LGCM, the multi-group growth model, the PLGCM, and the LGCM for two parallel processes. We also presented a description of a selection of the most commonly used statistical software programs available to estimate LGCMs. Our hope is to help researchers identify research questions that can be better handled through this flexible modeling framework.

## Cautions for all LGCM-based approaches

Model specification is an important issue within any sort of modeling framework (Curran et al., 2010). In the case of latent growth models, there are many features that one must be aware of when specifying the model. If, for example, the nonlinear function incorporated into the model is not representative of the patterns in the population, then substantive results may be impacted with this specification error embedded. Likewise, there is a part of the model that controls how related measures for the same subject are at different times, as well as how related (if at all) observations for different subjects are allowed to be. These elements in the model are controlled through within-individual and between-individual covariance matrices. The researcher can control whether time-points or people are allowed to covary through the manipulation of these matrices. Wu and West (2010) found that misspecification in the within-individual or the between-individual covariance structure can impact model fit statistics and change substantive conclusions. Therefore, it is always important to examine the specification of the model carefully and fully report the settings used to aid in interpretation of findings; see van de Schoot et al. (2017) for more details surrounding proper specification and reporting of latent growth models.

Applied researchers are often understandably concerned with whether a growth model specified actually fits the data patterns. For example, a researcher could specify a quadratic-shaped growth curve and examine whether it fits reasonably well compared to other growth shapes. In this case, a researcher would likely use model comparison measures (e.g., AIC or BIC) to make that assessment. One point of caution specific to growth models is that fit assessment does not just arise at the model-level. It is also possible to examine person-level fit, to see how well each individual's trajectory fits along with the specified model. One issue that can arise in nonlinear growth is that one form of nonlinearity (e.g., quadratic) may fit the full data best, but it could be that this growth shape is not what represents the bulk of individual growth trajectories. As an example, it is possible to have a quadratic model fit the full data set best, but have most of the individual trajectories follow a linear trajectory. In this case, there is a mismatch between overall and person-level fit with respect to the optimal model to select for interpretation. In this case, it is imperative to fully report findings and any discrepancy yielded. For more information on person-level fit, see Coffman and Millsap (2006).

## Conclusion

In summary, the LGCM and its various specifications is one tool in an array of quantitative methodologies for the study of change over time in biomarkers of the stress response. The choice of proper statistical methodology should be driven by a number of factors, including the research question, and the sample size. The methods described in this article provide a perspective that can be of great relevance to stress researchers. The purpose of this paper was to increase awareness of LGCMs and how they can be useful to stress researchers investigating biomarkers. The accessibility of more statistical methods permits the continued evolution and development of the types of research questions that can be asked.

## Author contributions

JF developed the idea, wrote the manuscript, and edited the manuscript. SD and JT participated in brainstorming and edited the manuscript.

### Conflict of interest statement

The authors declare that the research was conducted in the absence of any commercial or financial relationships that could be construed as a potential conflict of interest.

## Footnotes

^1^The online supplementary material can be found at: https://www.dropbox.com/sh/5hthz8ndfozyxyg/AACUZRwrSKOnqCbBhHeSGmTIa?dl=0
